# Male-biased genes are overrepresented among novel *Drosophila pseudoobscura *sex-biased genes

**DOI:** 10.1186/1471-2148-8-182

**Published:** 2008-06-24

**Authors:** Muralidhar Metta, Christian Schlötterer

**Affiliations:** 1 Institut für Populationsgenetik, Veterinärmedizinische Universität Wien, Wien, Austria

## Abstract

**Background:**

The origin of functional innovation is among the key questions in biology. Recently, it has been shown that new genes could arise from non-coding DNA and that such novel genes are often involved in male reproduction.

**Results:**

With the aim of identifying novel genes, we used the technique "generation of longer cDNA fragments from serial analysis of gene expression (SAGE) tags for gene identification (GLGI)" to extend 84 sex-biased 3'end SAGE tags that previously could not be mapped to the *D. pseudoobscura *transcriptome. Eleven male-biased and 33 female-biased GLGI fragments were obtained, of which 5 male-biased and 3 female-biased tags corresponded to putatively novel genes. This excess of novel genes with a male-biased gene expression pattern is consistent with previous results, which found novel genes to be primarily expressed in male reproductive tissues. 5' RACE analysis indicated that these novel transcripts are very short in length and could contain introns. Interspecies comparisons revealed that most novel transcripts show evidence for purifying selection.

**Conclusion:**

Overall, our data indicate that among sex-biased genes a considerable number of novel genes (~2–4%) exist in *D. pseudoobscura*, which could not be predicted based on *D. melanogaster *gene models.

## Background

Understanding functional innovation is one of the most interesting questions in biology. One important mechanism of functional innovation involves changes in gene expression [1] caused by cis-regulatory mutations [2]. While structural mutations within existing genes are an alternative mechanism to generate new functions [3], another possibility is the emergence of new genes. Several possible mechanisms are known to be involved in creating novel genes [4]. The best described origins of novel genes are gene duplication [5] and exon shuffling [6,7]. Recently it has been shown that novel genes could also originate *de novo *from non-coding regions [8]. Comparative genome analyses permit the identification of previously uncharacterized genes through sequence conservation, but the identification of rapidly evolving genes or genes of very recent origin is frequently restricted to *in silico *predictions. As novel genes are typically short [8,9], these may be easily missed. Alternatively, gene expression could serve as a good indicator for the presence of a gene. Hence, either Expressed Sequence Tag (EST) databases or reverse SAGE [10,11] could be used to identify novel transcripts.

Drosophila served as model for the identification of novel genes since the 1990s. One of first novel genes in this genus was *jingwei *in *D. melanogaster *[12], which is a fusion of two genes, a retroposed copy of the *alcohol dehydrogenase *(*Adh*) gene and a duplicated copy of the *yellow emperor *(*ymp*) gene [13]. Since then several studies applied phylogenetic methods to the growing databases aiming for the identification of novel genes. The majority of the novel genes have a sex-biased gene expression and some reports suggested that sex-biased genes change their expression pattern more rapidly than unbiased genes [14,15]. Furthermore, male-biased genes were shown to have a higher rate of protein evolution than unbiased genes [16-18]. In a recent report comparing the pattern of gene expression in *D. melanogaster *and *D. pseudoobscura *we failed to find evidence for an unconditionally faster rate of sequence evolution of male-biased genes. Rather, only genes with a male-biased gene expression in *D. melanogaster *were found to evolve faster. Genes with a male-biased gene expression in *D. pseudoobscura *only were evolving at a similar rate as unbiased genes [19]. As a large proportion of the sex-biased tags could not be mapped to the corresponding genes in *D. pseudoobscura*, the analysis of these tags should shed further light onto the pattern of protein evolution of sex-biased genes in *D. pseudoobscura*.

In this study, we identified eight novel genes with sex-biased gene expression in *D. pseudoobscura *using GLGI (Generation of longer cDNA fragments from serial analysis of gene expression tags for gene identification). Consistent, with previous results [8,9], we observed significantly more novel genes with a male bias than with a female bias in gene expression. Interestingly, we found no significant excess of X-linked novel genes, as has been reported in the previous studies [8,9].

## Results

### GLGI analysis

We used recently published SAGE data to identify sex-biased tags in *D. pseudoobscura *[19]. Previous analysis showed a substantially higher efficiency of tag to gene mapping for male-biased tags than for female-biased tags [19]. As the *D. pseudoobscura *genome annotation is heavily based on *D. melanogaster *gene models, this may be due to a higher proportion of novel genes among the genes with a female-biased gene expression. To test this, we selected 20 male-biased and 64 female-biased tags that were previously not mapped, relatively highly expressed and showed significant difference in expression between the sexes (*p *< 0.001), for further analysis. Using the GLGI method, we successfully generated longer 3'cDNA fragments for 44 SAGE tags. This success rate is in agreement with a previous GLGI analysis [20]. The GLGI fragments include 11 male-biased and 33 female-biased tags (Table 1). Thirty female-biased (91%) tags were mapped close to putative orthologs of *D. melanogaster *in the 3' end while only six male-biased (55%) tags were in this category (Additional file 1, Tables 1a and 1b). The remaining eight tags are falling into genomic regions that show no sequence conservation between *D. melanogaster *and *D. pseudoobscura *and no genes are annotated in *D. melanogaster*. Furthermore, we could not identify these regions by either nucleotide or protein BLAST search in any other species whose genome is completely sequenced except for *D. persimilis*. This suggests that these tags potentially represent novel genes that arose in the obscura group and are absent in other Drosophila species. Alternatively, these genes may have diverged from their orthologs in other Drosophila species to such an extent that they could not be identified by BLAST algorithm. We identified five male-biased and three female-biased transcripts that are putatively novel. Hence, 46% of the male-biased and 9% of the female-biased tags are putatively novel in the genome of *D. pseudoobscura*. The difference between male- and female-biased tags is significant (*p *= 0.02), suggesting that the majority of the novel genes show male biased expression [8,9].

**Table 1:** Overall statistics of tags used in this analysis

	Male biased	Female biased
Total tags for GLGI analysis	20	64
Successfully amplified tags	11	33
Putative novel genes	5	3
Successfully amplified novel genes using 5'RACE	5	1

### Modified SAGE-GLGI approach

As the GLGI analysis of sex-biased genes indicated a high number of novel genes, we were interested if this applies only to highly sex-biased genes or is a more general phenomenon. We sequenced 126 clones using a cDNA library that mimics the generation of GLGI fragments from SAGE tags. Of the 126 clones sequenced, 89 were unique and the remaining clones were redundant. From these 89 clones, 46 clones could be matched to the SAGE expression data [19]. Out of 89, 35 clones could be mapped to the annotated genes of *D. pseudoobscura*. Thirty-two clones mapped close to predicted genes in the 3' end. Six clones fell unambiguously into genomic regions for which genes are predicted in *D. melanogaster *but not in *D. pseudoobscura*. For these clones we noted that the incomplete annotation in *D. pseudoobscura *is probably due to duplications of the genes in these regions. One clone was mapped to a mitochondrial rRNA gene that is not included in the annotation of *D. pseudoobscura*. Three clones were missing in the *D. pseudoobscura *genome assembly but exist in the trace archive database [21] and have homologous sequences in *D. melanogaster*. Four clones were too short to be mapped unambiguously and two clones were anti-sense transcripts. For four clones of the six remaining ones, no prediction exists in either of the species despite sequence conservation. Hence, these genes are not novel to *D. pseudoobscura*. The remaining two clones are putatively novel genes as these sequences are located in genomic regions with no gene predicted in *D. pseudoobscura *and no orthologous region could be identified in *D. melanogaster *by a BLAST search. Both of these clones map to *D. pseudoobscura *chromosome arm XR. One of them matched a SAGE tag that was present in the male and female SAGE library [19] and showed female-biased expression. This sequence is located 72-nucleotides away from a predicted protein coding gene GA23511 in *D. pseudoobscura*. However, TBLASTN search of this protein against other completely sequenced Drosophila genomes did not reveal any hit except in the sibling species *D. persimilis*. The other clone did not match to any of the SAGE tags and was located in a region of the genome where no gene is predicted.

### Chromosomal distribution of novel sex-biased genes

Six of the eight GLGI sequences representing putatively novel genes are located on autosomes, while only two were located on the X-chromosome. Based on the number of predicted genes on X- and autosomes in the *D. pseudoobscura *annotation release 2.0, we tested for an overrepresentation of novel genes on the X-chromosome and did not find support for an enrichment of new genes on one chromosome (*p *= 0.72, Fisher's exact test). Even after accounting for the two novel genes identified by the modified GLGI approach, we still found no evidence for an enrichment of novel genes on the X-chromosome (*p *= 0.75, Fisher's exact test).

### Structure of the novel genes

**Figure 1:**
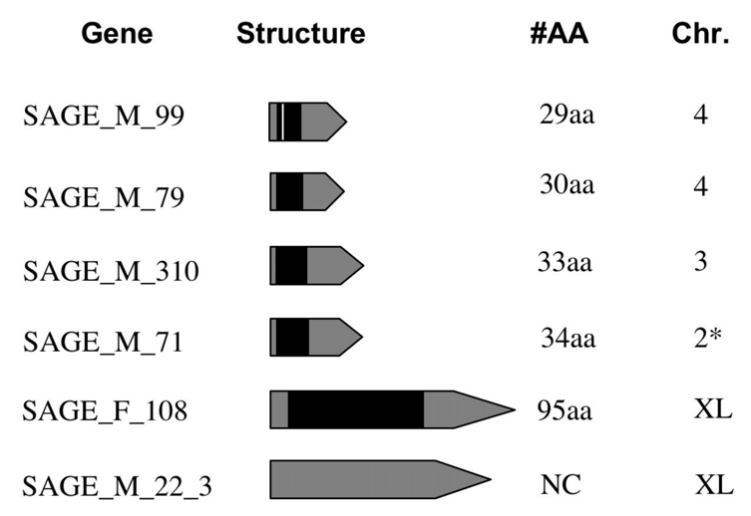
**Novel genes in *D. pseudoobscura***. List of novel genes obtained by analyzing SAGE, GLGI and 5'RACE with their protein length and chromosomal location. Black boxes represent coding regions, grey boxes represent non-coding regions and UTRs and white boxes represent introns. For the gene SAGE_M_71 (*), the chromosome location is determined based on its synteny in *D. persimilis*.

In order to obtain full-length transcripts of the novel genes identified by GLGI, we performed 5' RACE analysis. All the five male biased tags were successfully amplified with 5' RACE. Out of three female-biased clones, only one could be cloned and sequenced. Based on the 5' RACE analysis, we could show that the transcripts of these novel genes are very short, ranging from 138 base pairs to 442 base pairs, yielding very short coding sequences ranging from 29 amino acids to 95 amino acids (Figure 1). These transcripts have start and stop codons at the same position in the orthologous regions of the closely relatives *D. persimilis *and *D. miranda *and the complete ORF of these transcripts is conserved in the three species. Interestingly, the gene containing the shortest ORF was found to possess an intron. Nevertheless, further experiments are required to determine whether or not these short ORFs are translated into peptides. For one of the male-biased transcripts (SAGE_M_22_3), no potential coding sequence could be obtained and hence we assumed that it could be a non protein coding transcript.

### Cross-species conservation of novel genes

We took advantage of the 12 sequenced Drosophila genomes and performed TBLASTN searches with default parameters provided by flybase [22] to identify orthologs of the novel genes. Apart from *D. persimilis*, a very close relative of *D. pseudoobscura*, we did not detect any sequence similarity in the other species. Using PCR primers conserved between *D. pseudoobscura *and *D. persimilis *(See Additional file 1, Table 2), we successfully amplified the novel genes in *D. miranda*. Successful amplification of orthologs of the new, putatively protein coding genes from cDNA indicated their conservation in *D. miranda*. This implies that the novel genes arose before the split of *D. pseudoobscura *and *D. miranda*, which occurred about 2–5 MYA [23-25]. We assessed the protein coding potential of these short transcripts using the QRNA software, which distinguishes between coding alignments, conserved non-coding alignments and RNA secondary structure [26]. For none of the six transcripts did we find support for a protein coding potential (Table 2). However, as the sequences were short and the divergence between *D. miranda *and *D. pseudoobscura *is low, it is not clear how powerful this method is. Furthermore, it has been noted previously that failure of QRNA to identify a coding potential does not exclude the possibility of small ORFs [27]. Except for one (SAGE_M_79), all putatively protein coding genes had a ratio of synonymous to non-synonymous substitutions smaller than one, indicating purifying selection on a protein sequence. SAGE_M_79 contained only non-synonymous substitutions and no synonymous substitutions (Table 2). Supporting this hypothesis, the ratio of polymorphic non-synonymous substitutions to synonymous substitutions is also smaller than one for the polymorphic loci (Table 3). On the other hand, the likelihood ratio test (LRT), which tests if the dN/dS ratio is significantly lower than 1, was not significant for any of the loci except SAGE_M_71 (Table 2). However, it should be noted that the power of LRT is affected by sequence length, divergence and number of species [28] and may lead to a lower power in our case.

**Table 2:** Synonymous (dS) and non-synonymous (dN) ratios between *D. pseudoobscura *and *D. miranda *and QRNA predictions of the novel genes

Gene	dN	dS	dN/dS	**LRT**^#^	QRNA*
SAGE_M_99	0.029	0.063	0.462	0.570	O287
SAGE_M_79	0.045	0.000	-	0.208	O328
SAGE_M_310	0.014	0.036	0.394	0.270	O202
SAGE_M_71	0.013	0.134	0.097	0.049	O154
SAGE_M_22_3	-	-	-	-	O416
SAGE_F_108	0.024	0.056	0.430	0.102	R460

^#^Likelihood ratio test was performed using *D. pseudoobscura*, *D. persimilis *and *D. miranda*.

* O – other conserved non-coding sequences; R – RNA secondary structure. The number after the letter indicates the number of nucleotides assigned by QRNA to this category, which is in all cases equivalent to length of the full transcript.

**Table 3:** Neutrality tests for the novel genes in Mesa-Verde population (n = 8)

Gene	H	π	Tajima's D*	πN	πS	πN/πS
SAGE_M_71	6	0.012	0.687	0.0000	0.0725	0.000
SAGE_M_79	1	0.000	0.000	0.0000	0.0000	0.000
SAGE_M_99	5	0.006	-0.503	0.0061	0.0153	0.393
SAGE_M_310	1	0.000	0.000	0.0000	0.0000	0.000
SAGE_F_108	7	0.007	-0.414	0.0025	0.0155	0.163

H = Number of Haplotypes.

π = Nucleotide diversity.

* *p *> 0.05

### Evolution of novel genes

Recently evolved novel genes were shown to be under adaptive evolution in *D. melanogaster *lineage species [8,9]. To understand the pattern of variation and to test the deviations from neutral expectations in the novel genes of *D. pseudoobscura*, we sequenced the protein coding genes in one *D. pseudoobscura *population from Mesa-Verde (Colorado, USA, n = 8). Neutrality tests like Tajima's D (Table 3), Fu & Li's D, Fu & Li's F and Fu's Fs did not show a significant deviation from neutral expectations. We also performed a McDonald-Kreitman test [29] using *D. miranda*, which basically assumes that under neutrality, the ratio of fixed differences between species to polymorphic differences within species should be similar for both the synonymous and non-synonymous sites. None of the novel loci showed significant deviations from neutral expectations (Table 4). It should be noted, however, that due to the short sequence of the loci analyzed the statistical power to detect selection is limited. A multi locus HKA test [30], which tests for the deviations between the polymorphism within species versus divergence between species across different loci was also not significant, suggesting that none of the genes deviates significantly from the remaining ones.

**Table 4:** McDonald-Kreitman test contingency table for the novel genes

Gene	Fixed differences		Polymorphic differences	
		
	Synonymous	Non-synonymous	Synonymous	Non-synonymous
SAGE_M_71	2	2	0	4
SAGE_M_79	0	4	0	0
SAGE_M_99	0	2	1	1
SAGE_M_310	1	1	0	0
SAGE_F_108	5	4	3	1

## Discussion

In this report we showed that the extension of SAGE tags by GLGI is a powerful approach for the identification of novel genes.

### Origin of the novel genes

Novel genes can be generated through different evolutionary trajectories: retrotransposition [31], gene/genome duplication [5,32] and exon shuffling [7]. All these processes have in common that novel genes are built from existing genes or exons. Hence, it is expected that these building blocks should be detectable in the genome. We performed BLAST search of the novel genes against the *D. pseudoobscura *genome and detected only for one gene (SAGE_M_310) two BLAST hits. These two hits with complete sequence conservation were separated by 4.2-kilobase and the entire duplicated region spans approximately 1.1-kilobase with 92% identity. While we cannot rule out an assembly error, this observation suggests a recent duplication of the entire region encompassing the novel gene. Nevertheless, it is also apparent that both copies qualify as novel genes by our criteria.

Given that we lack support for the origin of the described novel genes from already existing ones, we favour the hypothesis that they are derived from previously non-coding regions. Recently, several cases of such novel genes derived from non-coding sequences were described in other Drosophila species [8,9]. Like in our study, the functional evidence came from gene expression in the focal species. In order to obtain additional support for functionality of these transcripts, we also tested for gene expression in a related species and found all of the novel genes also to be transcribed in *D. miranda*. But, we cannot exclude that these transcripts are non-coding or the regions are spuriously expressed in the closely related species. However, such small ORFs were previously reported in Drosophila and other species and are known to be functional [33-36]. The generation of novel genes by mutation appears a very unlikely event and the chance of generating a functional gene by random mutations decreases with the length of a gene. Hence, it is particularly interesting that all the novel genes identified in this study and previous ones [8,9] tend to be very short. Once more novel genes become available in combination with information about their date of origin, it will be possible to infer the probability to generate novel genes by random mutations. In particular, we will gain more insight if some sequences are more prone to develop into functional genes.

### Novel genes are male-biased

In this report we searched for novel genes among SAGE tags showing either a strong male or female expression bias. Despite that we screened a considerably larger number of female-biased SAGE tags, the majority of the novel genes had a male-biased gene expression pattern. This pattern is consistent with previous results [8,12,37-39]. It appears to be a general trend that many novel genes possess functional significance in males relative to females, but the reason for this trend is still not completely clear. Possible reasons include a higher transcription rate in male germline, greater functional pleiotropy of genes expressed in females and/or sexual competition [40]. Alternatively, there could be more testis specific promoter sequences in the genome.

### Non-preferential X chromosomal location of novel genes

There has been considerable controversy about the genomic location of male-biased genes. Earlier theoretical predictions [41] as well as expression studies in mice [39] suggested that recessive genes conferring an advantage to males should be located on the X-chromosome. Expression studies in Drosophila [42] and *C. elegans *[43] showed, however, that male biased genes are preferentially located on the autosomes. This discrepancy could be explained by X-chromosome inactivation during spermatogenesis [44-46]. The preferential X-linkage of novel genes, which show a male expression pattern [8] requires further explanation. It has been suggested that genes conferring an advantage to males first originate on the X-chromosome and move to an autosomal location later in evolution [42,47]. Hence, it is conceivable that these novel male-biased genes do not serve functions that are essential during male spermatogenesis. Once such novel genes become essential, they could move to the autosomes. It was also suggested that mutations generating *de novo *genes might occur more often on the X chromosome or fix more readily [8]. The novel genes we identified in this analysis can be dated from a minimum of 2–5 MYA (*D. pseudoobscura *and *D. miranda *divergence time [23-25]) to a maximum of 13–15 MYA (divergence of obscura group [25,48]). It is possible that the novel genes identified in this study may be older compared to the novel genes reported by Levine et al. [8], which was 2.5 MYA and if this is true, then these genes might have already moved to autosomes [42]. More data are required to see if the discrepancy between our study and the results of Levine et al. [8] are related to the difference in age or could be simply attributed to sampling effects due to the small number of genes in both studies.

### High incidence of novel genes in *D. pseudoobscura*

The discovery of previously uncharacterized transcripts is an observation common to many SAGE and EST sequencing experiments [11,39,49,50]. Nevertheless, the identification of evolutionary novelties requires also the absence of sequence conservation in related species. This approach was pioneered by Schmid and Tautz [51], who studied the conservation of cDNAs across various species using hybridization to genomic Southern blots. More recently, Levine et al. [8] specifically searched for lineage specific genes by BLAST search of all cDNA sequences available in *D. melanogaster *against *D. yakuba*, *D. erecta *and *D. ananassae*. A similar approach was also pursued for *D. yakuba *by BLAST search of ESTs against the genomic sequences of *D. yakuba*, *D. melanogaster*, *D. erecta *and *D. ananassae *[9]. In this study, we focused on novel genes in *D. pseudoobscura*. Our previous SAGE analysis indicated that about 12% of the genes surveyed showed a significant sex bias. Of these, 73% could not be mapped to the *D. pseudoobscura *transcriptome and the majority of them possessed female-biased gene expression. This discrepancy could arise from a high number of novel female-biased genes in *D. pseudoobscura*. Contrary to our expectations and in agreement with the other studies we observed a high number of male-biased novel genes compared to female-biased novel genes. This result could be the outcome of different rates of evolution of male and female-biased genes [15,18] in *D. pseudoobscura*.

In this study, we showed that 18% of the unmapped SAGE tags originate from putatively novel genes that arose in the *obscura *group. Hence, among the sex-biased genes approximately 2% are novel. In a cDNA library from *D. pseudoobscura *females we observed about 4% novel genes. This high incidence of novel genes in *D. pseudoobscura *underlines the need to supplement available genomic sequences with a thorough characterization of their transcriptome. In wake of the recent advances in the sequencing technology, we anticipate that this is already in close reach.

## Conclusion

We identified eight novel genes with sex-biased gene expression in *D. pseudoobscura *from unmapped SAGE tags using the GLGI method. In agreement with previous results from the other Drosophila species, a majority of these novel genes are male-biased in gene expression. Interestingly, we found no significant excess of X-linked novel genes. Overall, our data show that a considerable number of novel sex-biased genes exists in *D. pseudoobscura *that could not be predicted based on *D. melanogaster *gene models, which underlines the need to supplement available genomic sequences with a thorough characterization of their transcriptome.

## Methods

### GLGI analysis

*D. pseudoobscura *SAGE tags were obtained from Metta et al. [19]. The GLGI procedure for the 3' extension of the SAGE tags was performed according to Chen et al. [52]. In brief, total RNA was extracted separately from 15 male and 15 female virgin flies using Trizol (Invitrogen, Carlsbad, CA) and treated with DNAseI (MBI Fermentas) to digest the genomic DNA residues. PolyA mRNA was isolated using a 5' biotinylated and anchored oligo dT primer (5' biotin-ATCTAGAGCGGCCGC(T)_16_V) and streptavidin beads (Dynal). Double strand cDNA was synthesized using the "Double strand cDNA synthesis kit" (Invitrogen, Carlsbad, CA) and digested with the NlaIII isoschizomer Hin1II (MBI Fermentas). The 3' end fragments of the digested cDNA was recovered with Dynal beads. PCR amplification was performed using SAGE tag as 5' primer and anchoring sequence to the polyA was used as 3' primer. The PCR product was then cloned into TA vector (Invitrogen, Carlsbad, CA) and sequenced.

### Modified SAGE-GLGI method for detecting novel genes

To verify the consistency of the abundance of novel genes, we also performed an independent analysis, which is a modification of the SAGE-GLGI approach. We used mRNA isolated from virgin females. Double stranded cDNA was digested with NlaIII and an adapter (5'-GCCTCCCTCGCGCCATCAGCATG-3' and 5'-CTGATGGCGCGAGGGAGGC-3') was ligated to the 5' end of the digested cDNA fragments. The resulting template was amplified using an adapter specific primer and the primer anchoring to oligo dT. The products were cloned into TA vector and sequenced using M13 primers.

### Bioinformatics

BLASTN search was performed using the *D. pseudoobscura *database to identify the location of the GLGI (and modified SAGE-GLGI) fragments in the genome. As the *D. pseudoobscura *genome is only coarsely annotated we re-annotated the *D. pseudoobscura *genome for the regions of interest. We noted that a GeneWise analysis using the *D. melanogaster *gene model often results in a more complete protein prediction than the one available in the annotation database [22]. Hence we first performed GeneWise using those *D. melanogaster *genes that mapped in the proximity of the GLGI fragments to test if the *D. pseudoobscura *gene prediction may have been incomplete and the GLGI fragment matches to the extended protein coding region. As neither our GeneWise annotations nor the available annotation of *D. pseudoobscura *contained UTR sequences for most genes, we also used *ab initio *gene predictions. Specifically, we submitted the genomic region spanning the 5' part of the closest gene in the correct orientation and the genomic region matching to the GLGI fragment to a Genescan prediction. The coding potential of the transcripts was assessed using QRNA program [26].

### Structure of novel genes

To identify the 5' boundaries of the novel GLGI sequences, we performed 5' RACE (rapid amplification of cDNA ends) using GeneRacer RACE ready cDNA kit (Invitrogen, Carlsbad, CA). The 5' RACE analysis was performed on an independent extraction of total RNA.

### Evolution of novel genes

Eight individuals of *D. pseudoobscura *from Mesa-Verde (Colarado, USA) were sequenced for the five novel genes putatively coding for a protein (see Additional File 1, Table 2 for the primer sequences). Orthologous regions in *D. persimilis *were obtained from flybase [22]. One individual of *D. miranda*, a close relative of *D. pseudoobscura*, was obtained from the Tucson stock center (stock number: 14011-0101.08) and sequenced for these loci at both genomic as well as transcriptomic level. A typical cycling profile consisted of 3 min denaturation at 94°C followed by 35 cycles of 94°C for 40 sec, 50°C for 50 sec, and extension at 72°C for 1 min. PCR products were sequenced for both strands using DYEnamic ET Terminator Sequencing Kit (GE Health care Bio-Sciences AB, Sweden) according to manufacturer's instructions. The extension products were purified with Sephadex G-50 fine (GE Health care Bio-Sciences AB, Sweden) and separated on a MegaBACE 500 automated capillary sequencer. Forward and reverse strands were assembled using CodonCode Aligner version 2.0.1 [53]. The sequences were aligned using clustalW [54] and standard neutrality tests and McDonald-Kreitman test were performed using DnaSP version 4.10 [55]. The pair wise synonymous and non synonymous substitutions between *D. pseudoobscura *and *D. miranda *were obtained using yn00 program of PAML [56]. Likelihood ratio test was performed using codeml program of PAML using three species tree to test if the dN/dS ratio is significantly lower than 1. Multi locus HKA test [30] was performed using HKA program written by Jody Hey [57]. All the sequences were submitted to GenBank (accession numbers EU379026–EU379076).

## Abbreviations

SAGE: serial analysis of gene expression; GLGI: generation of longer cDNA fragments from serial analysis of gene expression tags for gene identification; RACE: rapid amplification of cDNA ends; UTR: untranslated region; ORF: open reading frame; MYA: million years ago.

## Authors' contributions

MM carried out the experiments, analyzed the data and drafted the preliminary manuscript. CS conceived of the study, and participated in its design and coordination and drafted the manuscript. All authors read and approved the final manuscript.

## Supplementary Material

Additional file 1 **1a – List of female-biased tags with successful GLGI amplification. 1b – List of male-biased tags with successful GLGI amplification**. **†**The gene is not predicted in the database of *D. pseudoobscura*. "Within the gene" indicates that the tag is located within the transcript but as it belongs to a mitochondrial gene or an unpredicted gene, it was not mapped previously. "Splice form" indicates that the SAGE tag is falling within an intron of a gene and so it is likely to be a splice variant. "Anti-sense" indicates that the GLGI sequence is matched in the anti-sense direction of the predicted gene. *This tag did not give any hit in *D. pseudoobscura *genome assembly but exists in the trace archive. The chromosome location is determined based on the sequence synteny in *D. persimilis*. "Sequencing artefact" indicates that the tag is falling within the gene but the region is having no sequence coverage (represented by 'n' in the database). **2 – Primers used for cross-species amplification as well as population analysis of *D. pseudoobscura*.** Click here for file
