# Supplementary material for: Male-biased genes are overrepresented among novel Drosophila pseudoobscura sex-biased genes
Source: BMC Evol Biol. 2008 Jun 24;8:182. doi: 10.1186/1471-2148-8-182 (PMC2443716; doi:10.1186/1471-2148-8-182)
Supplement: Additional file 1 — 1a – List of female-biased tags with successful GLGI amplification. 1b – List of male-biased tags with successful GLGI amplification. †The gene is not predicted in the database of D. pseudoobscura. "Within the gene" indicates that the tag is located within the transcript but as it belongs to a mitochondrial gene or an unpredicted gene, it was not mapped previously. "Splice form" indicates that the SAGE tag is falling within an intron of a gene and so it is likely to be a splice variant. "Anti-sense" indicates that the GLGI sequence is matched in the anti-sense direction of the predicted gene. *This tag did not give any hit in D. pseudoobscura genome assembly but exists in the trace archive. The chromosome location is determined based on the sequence synteny in D. persimilis. "Sequencing artefact" indicates that the tag is falling within the gene but the region is having no sequence coverage (represented by 'n' in the database). 2 – Primers used for cross-species amplification as well as population analysis of D. pseudoobscura. [file 1471-2148-8-182-S1.doc]

# Additional files

### Additional table 1a –

| Tag sequence | Name | Chr. | Expression | | Gene | Distance from 3’UTR |
| --- | --- | --- | --- | --- | --- | --- |
|  |  |  | Male | Female |  |  |
| GCGGAGACAA | SAGE_203 | 2 | 65 | 203 | GA15543 | 70nt |
| TAAGAATTTA | SAGE_143 | 2 | 20 | 143 | GA20384 | 127nt |
| TGTACAGACA | SAGE_108 | XL | 40 | 108 | New |  |
| AATGAAATCA | SAGE_84 | Mt | 40 | 84 | mt coxII | Within the gene |
| TATGTGTTTG | SAGE_49_2 | U | 2 | 49 | Ortho_CG5271† | 31nt |
| AGCAAACCTA | SAGE_47 | XL | 21 | 47 | GA20486 | 51nt |
| AGGTTATATC | SAGE_38 | XL | 9 | 38 | Ortho_CG4918† | Within the gene |
| AAATCACGAA | SAGE_34_13 | XR | 13 | 34 | GA17760 | Splice form |
| GCCGGGGTAG | SAGE_29_2 | 4 | 2 | 29 | GA21667 | Splice form |
| TAAGCCGAAA | SAGE_27_3 | XL | 3 | 27 | GA15195 | 224nt |
| CAAAACTCAT | SAGE_26_3 | 2 | 3 | 26 | GA15307 | 495nt |
| TCTAAAAACA | SAGE_26_6 | 2 | 6 | 26 | GA21118 | 98nt |
| TTTGAATAAC | SAGE_25_2 | 3 | 2 | 25 | GA16529 | 144nt |
| CAATTTCCTT | SAGE_24_1 | XL | 1 | 24 | GA15553 | 118nt |
| TTTTATGATA | SAGE_21_2 | XL | 2 | 21 | GA12822 | 110nt |
| CAAAAAGATC | SAGE_19_3 | 2 | 3 | 19 | GA17647 | 78nt |
| TACGCGATTC | SAGE_18_2 | XL | 2 | 18 | GA13593 | 91nt |
| CGGATTTGAT | SAGE_18_4_CGG | 3 | 4 | 18 | GA21061 | 247nt |
| AAAAATTAAC | SAGE_16_0_AAA | 3 | 0 | 16 | GA13509 | 36nt |
| TTGGCGTCTA | SAGE_16_0_TTG | 2 | 0 | 16 | GA21118 | Anti-sense |
| GCACCGAACA | SAGE_16_4 | 4 | 4 | 16 | GA14525 | Anti-sense |
| GGCGTTGCAA | SAGE_14_1_GGC | 2 | 1 | 14 | GA13070 | 142nt |
| TAAACAGTGA | SAGE_121 | 4 | 40 | 121 | GA18510 | 42nt |
| CAAACATAAA | SAGE_85 | 3 | 24 | 85 | GA13467 | 28nt |
| AAAAGATCCA | SAGE_113 | XL | 44 | 113 | GA21411 | 110nt |
| AGCTGGATAA | SAGE_162 | 2 | 64 | 162 | GA15097 | 89nt |
| CAAATGGGAG | SAGE_309 | 4 | 129 | 309 | New |  |
| CAGCACCTAT | SAGE_151 | 2 | 84 | 151 | GA21812 | 91nt |
| CGAAATAAAG | SAGE_228 | 3 | 38 | 228 | GA20995 | 42nt |
| CGGAGCTGTT | SAGE_272 | XR | 96 | 272 | GA11782 | 22nt |
| GCGGACGTTA | SAGE_310 | 4 | 68 | 310 | New |  |
| TCTTTTTGAA | SAGE_140 | mt | 6 | 140 | mt 16s rRNA | Within the gene |
| TTAGGCATAC | SAGE_98 | XL | 1 | 98 | GA10778 | 30nt |

### Additional table 1b –

| Tag sequence | Gene Name | Chr. | Expression | | Gene | Distance from 3’UTR |
| --- | --- | --- | --- | --- | --- | --- |
|  |  |  | Male | Female |  |  |
| ATCCTGAAAT | SAGE_M_14 | 4 | 14 | 7 | GA14168 | Sequencing artifact |
| GAGTTCACGT | SAGE_M_139 | U | 139 | 119 | mt COXIII | Within the gene |
| CGGACTGCAG | SAGE_M_99 | 4 | 99 | 29 | New |  |
| GCAACTGGTA | SAGE_M_79 | 4 | 79 | 19 | New |  |
| TCTTCTCACA | SAGE_M_71 | 2* | 71 | 33 | New |  |
| CAGATCTCGG | SAGE_M_63 | 3 | 63 | 15 | GA11112 | 99nt |
| ACGATGTATG | SAGE_M_35_7 | 4 | 35 | 7 | Ortho_CG31872 | 57nt |
| ACGATGTGAG | SAGE_M_27_4 | 4 | 27 | 4 | Ortho_CG18284 | 47nt |
| CCTTAGACGT | SAGE_M_22_3 | XL | 22 | 3 | New |  |
| GAGCTGTATC | SAGE_M_46 | XR | 46 | 15 | Ortho_CG18628 | Within the gene |
| TCCCCGAAGA | SAGE_M_310 | 3 | 908 | 310 | New |  |

### Additional table 2 –

| Name | Primer sequence (5’ to 3’) |
| --- | --- |
| SAGE_M_99_F | ACTGTTGTAATAGCCCA |
| SAGE_M_99_R | TTCAAGCTTACGTGGCA |
| SAGE_M_79_F | GAAGATGAGCATATTAGT |
| SAGE_M_79_R | TCAAATTTATTATCACGA |
| SAGE_M_310_F | CACTCGTATTCATCGATCAC |
| SAGE_M_310_R | TTGATCTAGATTTCTGCAAG |
| SAGE_M_71_F | GAAGATCTTCGCTTGTGTG |
| SAGE_M_71_R | GGAATTTATTTCATCGTAT |
| SAGE_F_108_F | CATTCAGTCTACAAAAACC |
| SAGE_F_108_R | CTTCAATAAGAACAAGCTC |
